# Dietary mimicry of melena following chocolate cookie ingestion in a patient with colostomy: a case report

**DOI:** 10.1186/s13256-026-06172-y

**Published:** 2026-07-08

**Authors:** Clement P. Buclin, Nils Bürgisser, Gabriele Lombardo, Roxane Naimi

**Affiliations:** 1 Department of General Internal Medicine, Geneva University Hospitals, Rue Gabrielle-Perret Gentil 4, 1205 Geneva, Switzerland; 2 Faculty of Medicine, Geneva University, Geneva, Switzerland; 3 Department of Rehabilitation and Internal Medicine, Geneva University Hospitals, Geneva, Switzerland

**Keywords:** Case report, Gastrointestinal hemorrhage, Melena differential diagnosis

## Abstract

**Background:**

Melena is dark, tarry stools that typically indicate upper gastrointestinal bleeding. However, various dietary substances can alter stool color and potentially lead to diagnostic confusion. We present a rare case of stool discoloration caused by the ingestion of chocolate sandwich cookies.

**Case presentation:**

A Black man in his 30s with a transverse colostomy presented with sudden-onset dark, tarry stool, raising concern for melena. Initial management included intravenous proton pump inhibitors and consideration of urgent endoscopy. However, the patient’s laboratory results and overall clinical presentation were inconsistent with upper gastrointestinal bleeding. Upon further questioning, he disclosed having recently consumed four packets of chocolate sandwich cookies. These cookies contain alkalized cocoa, which is rich in dark pigments that are poorly absorbed. We hypothesized that the stool was colored by the cookies and successfully reproduced the observed effect by having the patient eat the cookies a second time which led to another episode of dark black stool.

**Conclusion:**

Awareness of dietary mimics of melena is crucial to avoid diagnostic errors and unnecessary interventions. This case highlights the importance of a thorough dietary history and the need for clinical caution before initiating treatment for presumed gastrointestinal bleeding in hemodynamically stable patients.

## Introduction

Melena refers to black, tarry stools that typically indicate upper gastrointestinal bleeding, usually originating proximal to the ligament of Treitz, though it can occasionally arise from bleeding in the distal small intestine [1]. In rarer cases, bleeding from the proximal colon may also result in melena-like stools [2, 3].

Upon presentation of melena-like stool and suspicion of upper gastro intestinal bleeding, both American and European guidelines recommend the use of Glasgow-Blatchford score to stratify pre-endoscopic risk of rebleeding and address patient either to emergent or outpatient endoscopy [4–6]. Pre-endoscopic management relies on hemodynamic resuscitation, restrictive blood transfusion, and interruption of antithrombotic agents [5, 6]. Despite being widely used, there is no clear evidence on proton pump inhibitor’s efficacy in preventing rebleeding, reducing need for surgery or lowering mortality [7, 8]. They may still be considered, according to society guidelines, to reduce the severity of endoscopic stigmata and thereby minimize the need for endoscopic intervention.

## Case presentation

A Black man in his 30s was admitted to the rehabilitation and internal medicine department for the management of a stage III pressure ulcer.

He was paraplegic since over a year in the context of a traumatic spinal cord injury caused by a gunshot wound. His complication included a neurogenic bladder requiring intermittent self-catheterization and neurogenic bowel dysfunction with fecal incontinence, for which a transverse colostomy had been performed. Beside painkillers, anti-spasmodic and anti-depressant therapy by amitriptyline 10 mg once daily, the patient benefited from thromboprophylaxis treatment with subcutaneous enoxaparin 40 mg. Additionally, he had received ibuprofen 400 mg three times daily for five days after admission to manage pressure ulcer-related pain. Notably, he did not receive any iron supplementation or bismuth-containing medications during his hospital stay.

About a week after admission, he presented two episodes of black tarry stools within 12 hours. Throughout this time, his vital signs remained completely normal and he experienced no abdominal pain, nausea or vomiting.

### Investigations

Control blood tests had been performed the morning before the presentation of the melena-like stools and served as comparison. Blood count showed a stable hemoglobin and thrombocytes whereas blood chemistry showed no elevation of blood urea nitrogen nor dissociation of the urea–creatinine ratio.

Upon presentation of two episodes of melena-like stools over a short period of time, the first suspected diagnosis was upper gastrointestinal bleeding. This hypothesis was supported by two important drug-related risk factors present in our patient. First, association between non-steroidal anti-inflammatory drugs (NSAID) and nonvariceal upper gastrointestinal has been widely documented [9]. Second, the patient was taking amitriptyline, an intermediate affinity serotonin selective recapture inhibitor, a drug category for which there is a growing body of evidence pointing towards an association with upper gastrointestinal bleeding [10, 11].

However, both the clinical presentation and the laboratory results contradicted this hypothesis. Apart from odorless black stool, our patient presented none of the classical sign and symptoms notably no epigastric pain and no hemodynamic repercussion with neither tachycardia nor hypotension [12]. On laboratory, hemoglobin was stable arguing against a severe upper gastrointestinal bleeding. Furthermore, the blood urea nitrogen to creatinine ratio, which can become elevated in upper gastrointestinal bleeding reflecting digestion of red blood cells and production of urea nitrogen, was normal in our patient [13, 14]. Our patient had a Glasgow-Blatchford score of 2: one point for the presence of melena and one point for a hemoglobin level of 126 g/L, which falls below the threshold for males in this scoring system. This placed him in the low-risk category for adverse outcomes, with an estimated 3% risk of rebleeding or other complications and supported non-urgent endoscopy [15, 16].

Not all black stools represent true melena. Various non-bleeding-related causes of dark stool coloration have been documented, including medication such as oral ferrous supplementation or bismuth, commonly used in Helicobacter pylori eradication therapies [17, 18]. In addition, certain foods like artificial food dyes or pork blood soup have been reported to mimic melena and lead to diagnostic confusion [19, 20]. Given that none of the medication dispensed during his hospitalization are known to produce dark stool, further anamnesis was then conducted with our patient to investigate any over the counter medication intake or dietary change in the 24 hours leading to the melena-like stool. The history revealed no medication but led the patient to spontaneously disclose that he had eaten four packages of around 150 g of a famous brand of dark black chocolate sandwich cookies.

Given that we were not able to find any academic source describing a dark coloration of stool upon consumption of chocolate sandwich cookies but that a google search using the terms “cookies” and “black poop” revealed several internet forums with users describing a similar experience, we proposed our patient to take part in a diagnostic rechallenge experiment. Upon obtaining his full informed consent, he was asked to consume four packages of the same brand of cookies and instructed to eat them at a similar pace as the previous time. No other food was consumed during that rechallenge to reproduce the exact conditions which led to the previous occurrence.

**Fig. 1:**
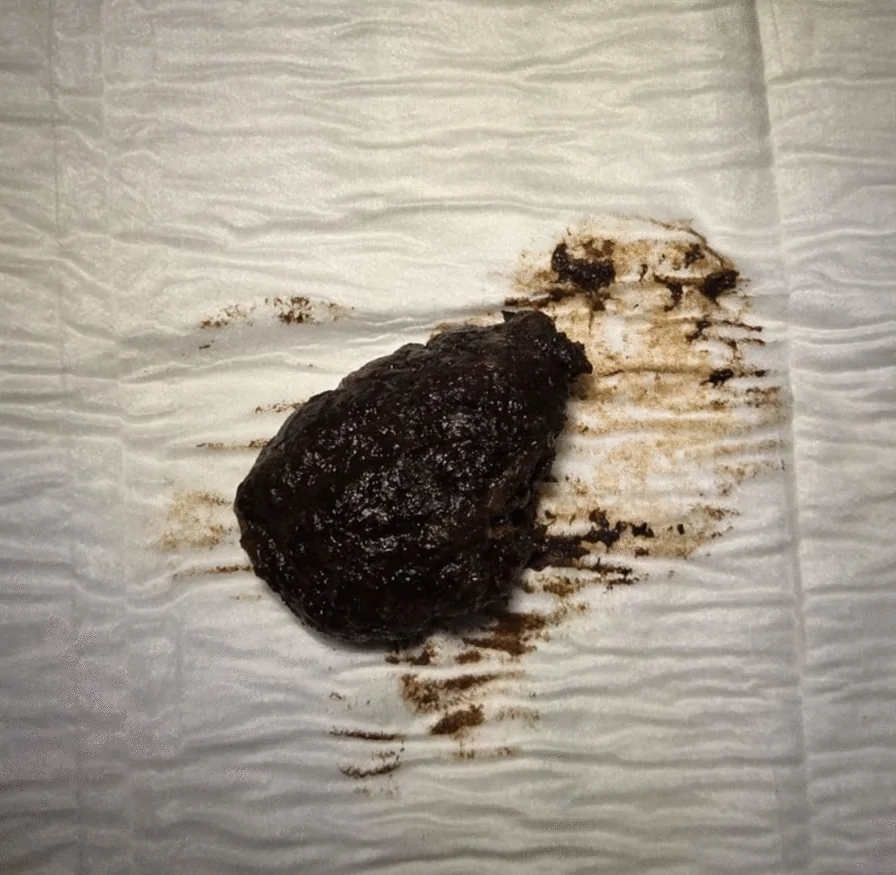
Stool sample 12 hours after chocolate sandwich cookie ingestion. The stool was tested using a fecal immunochemical test for occult blood and was negative. Picture is published with patient’s consent

12 hours after the start of this experiment, dark stools were passed in his colostomy bag. A sample of the stool was then visually inspected by all coauthors and photographed for documentation (Fig. 1). No stool color chart was used as they are not routinely used in our hospital. A fecal occult blood test was then performed on this sample and came back negative.

### Treatment

Initial treatment was started by intravenous proton pump inhibitors with esomeprazole 40 mg twice daily. Given hemodynamic and hemoglobin stability, treatment was interrupted after 36 hours.

### Outcome and follow-up

Esophagogastroduodenoscopy was originally considered to formally exclude an upper gastrointestinal bleeding before being definitively abandoned once the chocolate sandwich cookie experiments successfully reproduced black stools. One week after this episode, the patient has not passed any further black stool and his hemoglobin and blood urea nitrogen remained stable.

## Discussion

To our knowledge, no scientifically documented cases exist of chocolate sandwich cookie ingestion mimicking melena, though anecdotal recognition of this effect is common in online forums. Previous causes of dietary induced dark stools have been documented in patients consuming industrially dyed food like a black burger bun or pork blood soup [19, 20]. In the present case, the most likely scenario is that the ingestion of four packets of these cookies led to strikingly dark output from a patient with a transverse colostomy, raising clinical suspicion of melena.

While a definitive determination of the metabolic mechanism causing this stool coloration in our patient cannot be reached without further molecular research, previous food science research offers credible explanations. Like many other cookie brands, the famous brand of chocolate sandwich cookies consumed by our patient uses an industrial process called “dutching" to alkalize cocoa beans in order to give them a dark coloration and reduce the bitterness of cocoa [21–23]. Consumed in large quantities, these pigments would likely produce dark stool in any individual. In our patient, the presence of the colostomy may have made this effect more immediately visible rather than diluted within formed stool.

Additional mechanisms may have contributed. Cocoa beans contain large molecules such as procyanidins which absorption is mostly performed in the colon through microbiota action [24, 25]. In the context of a shortened colon, excessive intake of alkalized cocoa-containing cookies may have exceeded the absorptive capacity, potentially leading to further pigmentation of the stool and a misleading appearance of melena.

This misinterpretation prompted the administration of high-dose intravenous proton pump inhibitors (PPIs), which carry potential risks including acute interstitial nephritis and severe hypomagnesemia [26, 27]. The patient was also considered for esophagogastroduodenoscopy, a procedure with inherent risks, including bleeding and perforation in up to 0.5% of cases [28]. However, his Glasgow-Blatchford score of 2 placed him in a low-risk category where outpatient management is appropriate. This scoring system, combined with clinical judgment, supported a conservative approach and helped avoid unnecessary endoscopy.

Given the diagnostic weight placed on suspected melena in both institutional protocols and clinical scoring systems such as the Glasgow-Blatchford score, accurate recognition is essential. Previous attempts to standardize stool color interpretation, such as through color reference charts, have shown promise but have not been widely implemented or systematically validated [29, 30].

Fecal occult blood testing (FOBT) can aid in the evaluation of melena-like stools, but results must be interpreted with caution. Guaiac-based tests detect the heme and are generally positive in cases of true melena, whereas fecal immunochemical tests (FIT) detect the globin component, which is degraded by proteolytic enzymes during upper gastrointestinal transit [31]. Indeed, ingestion of up to 100 mL of blood may not yield a positive FIT result [32].

In this case, a fecal blood test was performed and returned negative. However, its diagnostic utility was limited, as our institution exclusively uses FIT for occult blood testing. As such, the result could not reliably exclude upper (gastrointestinal) GI bleeding and was informative only when considered alongside the patient’s clinical stability, low Glasgow-Blatchford score and normal laboratory findings.

## Conclusion

This case adds to the growing body of evidence on dietary and pharmacologic causes of melena-like stool by describing chocolate sandwich cookies induced darkened stool in a patient with a colostomy. It underscores the importance maintaining a broad differential and ensuring detailed history before proceeding with invasive investigations, particularly in hemodynamically stable patients.

## Data Availability

Not applicable.
